# Supplementary material for: Adsorption of Hexavalent Chromium by Sodium Alginate Fiber Biochar Loaded with Lanthanum
Source: Materials (Basel). 2021 Apr 26;14(9):2224. doi: 10.3390/ma14092224 (PMC8123644; doi:10.3390/ma14092224)
Supplement: Supplementary file 1 [file materials-14-02224-s001.zip › materials-1067537-supplementary.pdf]

materials

MDPI

Supplementary Material

Adsorption of Hexavalent Chromium by Sodium Alginate Fiber Biochar Loaded with Lanthanum

Xinzhe Sun [1], Peng Guo [2], Yuanyuan Sun [1,*] and Yuqian Cui [1,*]

[1] College of Environmental Science and Engineering, Qingdao University, Qingdao 266071, China; 2018025497@qdu.edu.cn

[2] School of Chemical Engineering, China University of Petroleum (East China), Qingdao 266580, China; guopeng@upc.edu.cn

* Correspondence: cuiyq@qdu.edu.cn (Y.C.); sunyy@qdu.edu.cn (Y.S.); Tel.: +86 13793235399 (Y.C.); +86 15264230805 (Y.S.)

1. Text S1: Langmuir and Freundlich isotherm models

Langmuir adsorption isothermal model: $\frac{C_e}{q_e} = \frac{C_e}{q_m} + \frac{1}{bq_m}$, (1)

Freundlich adsorption isothermal model: $\log q_e = \frac{1}{n}\log C_e + \log K_f$, (2)

Where q_e (mg/g) represents the adsorption amount of the adsorbents per unit with the adsorbent at adsorption equilibrium, C_e (mg/L) represents the concentration of the adsorbents at adsorption equilibrium, q_m (mg/g) represents the maximum adsorption capacity of the adsorbents, and b represents the adsorption coefficient and the affinity strength between adsorbents and adsorbents. In addition, K_f is the adsorption capacity constant, indicating the adsorption capacity of the adsorbent. The n value may reflect the heterogeneity of the adsorbent or the adsorption reaction strength.

2. Text S2: Pseudo-first- and pseudo-second-order kinetic models

Pseudo-first-order kinetic equation: $\ln(q_e - q_t) = \ln q_e - k_1 t$, (3)

Pseudo-second-order kinetic equation: $\frac{t}{q_t} = \frac{1}{k_2 q_e^2} + \frac{t}{q_e}$, (4)

Where q_e and q_t (mg/g) are the amount of Cr (VI) adsorbed on the adsorbent at equilibrium at any time t (min), respectively. k_1 and k_2 are the separate rate constants for the pseudo-first-order and pseudo-second-order sorption models.

3. Text S3: Absorption thermodynamics

Thermodynamic formula: $\ln K_D = \frac{\Delta S}{R} - \frac{\Delta H}{RT}$, (5)

The thermodynamic parameter of adsorption is the Gibbs free energy change（ΔG，$kJ\cdot mol^{-1}$,), the enthalpy change（ΔH，$kJ\cdot mol^{-1}$）and the entropy change（ΔS，$J\cdot mol^{-1}\cdot K^{-1}$）. The thermodynamic equation of adsorption reaction reflects the change of the adsorption heat and temperature. R represents the ideal gas constant, of which the value is 8.314 $J\cdot mol^{-1}\cdot k^{-1}$; T is the thermodynamic temperature (K); and K_D ($L\cdot g^{-1}$) represents the adsorption equilibrium constant, which can be calculated by the Freundlich model.

The linear fitting of $\ln K_D$ and 1/T resulted in a line, and the enthalpy and entropy changes of the adsorption reaction were calculated by its slope and interception. In general, if ΔG< 0, the adsorption reaction is spontaneous; if ΔG >0, it is a spontaneous reac-

tion, and it can undertake the reverse. If ΔH >0, the adsorption reaction is endothermic reaction; if ΔH< 0, the adsorption reaction is an exothermic reaction; if ΔS >0, that is, the adsorption reaction of entropy, said confusion degree increases the adsorption system, and is advantageous to the spontaneous reaction.

Table S1. Physical properties of the different samples.

Samples	Surface Area (m^2/g)	Pore Volume (cm^3/g)		D_P (nm)
	S_{BET}	V_{toal}	V_{micro}	
700°CLa-BC	8.21	0.0037	0.0169	10.9198
800°CLa-BC	38.61	0.0328	0.0196	4.0095
900°CLa-BC	177.41	0.2515	0.0803	6.2749
1000°CLa-BC	52.08	0.0547	0.0255	5.2147

Table S2. Parameters of the Cr (VI) adsorption isotherms based on the Freundlich and Langmuir models.

Materi-als	Langmuir Model			Freundlich Model		
	q_m (mg/g)	b (L/mg)	R^2	K_f	n	R^2
700 °C La-BC	41.4	0.0041	0.976	0.505	1.543	0.929
800 °C La-BC	67.4	0.0049	0.953	2.255	2.098	0.950
900 °C La-BC	104.9	0.0037	0.962	1.715	1.716	0.964
1000 °C La-BC	49.9	0.0042	0.994	0.776	1.655	0.963

Table S3. Parameters of the Cr (VI) adsorption kinetics based on the pseudo-first-order and pseudo-second-order dynamic models.

Materi-als	Pseudo-first-order model			Pseudo-Second-Order Model		
	q_e (mg/g)	$K_1 \times 10^{-2}$	R^2	q_e (mg/g)	$K_2 \times 10^{-2}$	R^2
700 °C La-BC	14.3	15.19	0.9944	14.26	0.95	0.9880
800 °C La-BC	20.4	24.51	0.9696	20.42	0.40	0.9880
900 °C La-BC	32.4	10.96	0.9698	32.39	1.33	0.9998
1000 °C La-BC	15.5	15.21	0.9428	15.49	2.94	0.9995

Table S4. Thermodynamic parameters for chromate adsorption with La-BC (900 °C).

Temperature (K)	Thermodynamic Parameters		
	ΔH (KJ/mol)	ΔS (J/(mol·K))	ΔG (KJ/mol)
298	41.66	144.6	-1.44
308			-2.78
318			-4.059

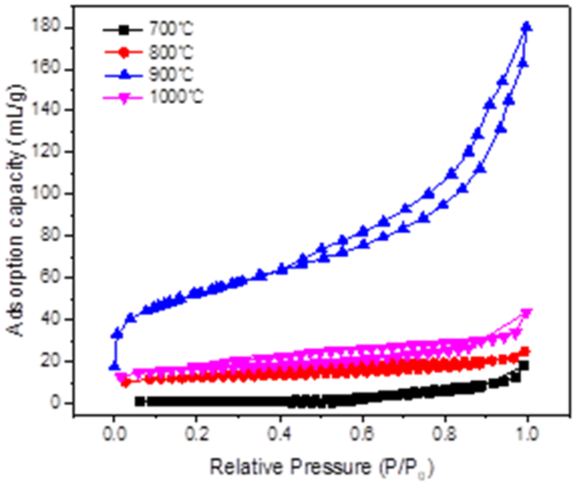

Figure S1. Nitrogen adsorption/desorption isotherms for different samples.

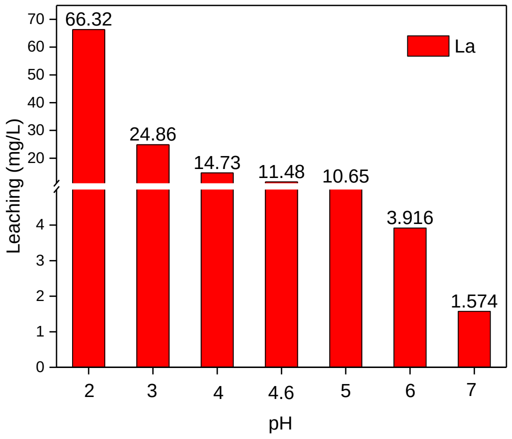

Figure S2. The leaching rate of lanthanum when the pH is in the range 2–7.
